# Antimicrobial activity of Titanium dioxide and Zinc oxide nanoparticles supported in 4A zeolite and evaluation the morphological characteristic

**DOI:** 10.1038/s41598-019-54025-0

**Published:** 2019-11-25

**Authors:** Maryam Azizi-Lalabadi, Ali Ehsani, Baharak Divband, Mahmood Alizadeh-Sani

**Affiliations:** 1 Students’ Research Committee, Department of Food Sciences and Technology, Faculty of Nutrition and Food Sciences, Tabriz University of Medical Sciences, Tabriz, Iran; 2 Nutrition Research Center, Department of Food Sciences and Technology, Faculty of Nutrition and Food Sciences, Tabriz University of Medical Sciences, Tabriz, Iran; 3 Dental and Periodontal Research Center, Tabriz University of Medical Sciences, Tabriz, Iran; 4 Students’ Scientific Research center, Food Safety and Hygiene Division, School of Public Health, Tehran University of Medical Sciences, Tehran, Iran; 5 Department of Food Sciences and Technology, Faculty of Nutrition and Food Sciences, Tabriz University of Medical Sciences, Tabriz, Iran; 6 Research Center for Environmental Determinants of Health (RCEDH), Kermanshah University of Medical Sciences, Kermanshah, Iran; 7 Food and Drug safety research center, Tabriz university of medical science, Tabriz, Iran; 8 Inorganic Chemistry Department, Faculty of Chemistry, University of Tabriz, C.P. 51664 Tabriz, Iran

**Keywords:** Nanoscience and technology, Nanotoxicology, Antimicrobials

## Abstract

In this study, the antimicrobial activity of titanium dioxide (TiO_2_), zinc oxide (ZnO), and TiO_2_/ZnO nanoparticles supported into 4A zeolite (4A z) was assessed. Based on antimicrobial experiments, minimum inhibitory concentration (MIC_90_), minimum bactericidal concentration (MBC), fractional inhibitory concentration (FIC) and disc diffusion test were determined after 24 h of contact with the prepared nanocomposites. These results are in agreements with the results of disc diffusion test. During the experiments, the numbers of viable bacterial cells of *Staphylococcus aureus*, *Pseudomonas fluorescens*, *Listeria monocytogenes* and *Escherichia coli* O_157_:H_7_ decreased significantly. The crystallinity and morphology of nanoparticles were investigated by X-ray diffraction patterns (XRD), elemental mapping at the microstructural level by scanning electron microscopy (SEM) with energy dispersive X-ray spectrometry (EDS), and transmission electron microscopy (TEM). As a result, it was demonstrated that TiO_2_/ZnO nanoparticles supported in 4A zeolite could lead to an optimum activity as antimicrobial agents.

## Introduction

Zeolites are microporous, alumino silicate minerals with open three-dimensional framework structures, made up of SiO_4_ and AlO_4_ tetrahedrons linked by sharing oxygen atoms to form regular intra crystalline cavities and channels of atomic dimensions. Zeolites are commonly used as commercial adsorbents and composites, as well as cation exchangers, molecular sieves and antimicrobial agents^1–3^.

Attaching nanoparticles on solid surfaces has been used as a traditional procedure to prepare heterogeneous composites with specific properties. Leaching and aggregation of nanoparticles often lead to composite deactivation^4,5^. In order to overcome these problems, some methods have been proposed including strengthening the metal-support interaction^6,7^ and using booster and setting out the morphology of nanoparticles^8,9^. Based on these advances, nanoparticles can be loaded on zeolites for antimicrobials, anti-fungal and anti-virus applications^10,11^. These achievements convinced researchers to produce nanoparticles inside zeolite. The new composite (zeolite with nanoparticles) can be tuned through the presence of precise nanopores^3,12,13^.

The issue of contamination by certain microorganisms in some materials, especially food, enables a significant hazardous pathway for humans. The most important pathogenic bacteria in food for mankind are *Salmonella spp, Escherichia coli O*_157_*H*_7_*, Staphylococcus aureus*, and *Listeria monocytogenes*^3,14^. Metal oxide nanoparticles (MONP) were tested with an excellent antimicrobial activity in the efficient removal of pathogens. MONP may not show a considerable antimicrobial activity in form of metal oxide and metal salt alone, because of their tendency of aggregation^15^. However, the stability and slow release of metal ions from MONP are effective properties that can be tuned by appropriate synthesis. For this reason, new methods to immobilize MONP into zeolite membranes have been suggested^16^. One of the most important factors in antimicrobial activity is light. MONP, particularly ZnO and TiO_2_, become activated against pathogenic bacteria when exposed to ultraviolet (UV) light^17,18^. The mechanism of this procedure involves substrate adsorption in the composite surface, depending on pH, temperature, composite stability, area and substrate concentration^19–22^. Some of the disadvantages are low adsorption ability (small active area) and photo corrosion^23,24^. Therefore, with precise preparation of ZnO and TiO_2_, especially regarding morphologies and sizes, most of these caveats can be overcome^25–27^.

MONP also show significant antimicrobial activity through connection to microbial DNA and proteins, and caused to preventing bacterial duplication, avoiding metabolic enzymes of the bacterial electron transport chain, leading to their inactivation^2^.

The semiconductor properties of zinc oxide (ZnO) and titanium dioxide (TiO_2_) can be tuned in nano scale^28–31^. Manufacturing nanoparticles involves protocols of different complexities with aggregation of ZnO and TiO_2_ nanoparticles as an important limitation in the process^32,33^. These compounds are useful in pharmaceutical utilization^34^, pigmentation^35^, antimicrobial activities, anti-fungal and anti-viruses properties^36^. In addition, ZnO and TiO_2_ are important components in the novel and active packaging industry.

Tiny ZnO and TiO_2_ nanoparticles with a wide surface can maintain their photoactivity; however, their recovery at the end of the procedure is very difficult. Accordingly, a useful approach is to support ZnO and TiO_2_ in a zeolite structure which can enhance adsorption and subsequently, increase the composite efficiency and ease of recovery by ordinary deposition^37,38^. The use of effective composites made up with ZnO and TiO_2_ impregnated in zeolites is a new strategy to produces nanoparticles with supporter. Recent research reported some properties of ZnO supported in zeolites including ZSM-5, Y, X and A^39,40^. In this research, we have focused on the description and development of ZnO and TiO_2_ nanoparticles supported in the channels of a porous matrix (4A zeolite) for antimicrobial application. First of all, we described a simple synthetic route for preparation of ZnO and TiO_2_ impregnated into 4A zeolite (4A z). Secondly, the structural properties and antimicrobial effects of the materials were determined. In fact, zinc and titanium in high quantities may have toxic effects^41–45^; hence, in this work we have tried to use 4A z as a support of metal nanoparticles. These nanoparticles are encapsulated inside zeolite cavities or external channels to decrease the possibility of leaching as low as possible. This process reduces the toxicity of nanoparticles and their release from the matrix and therefore, this complex (nanoparticles and zeolite) can be used in polymer matrix for food packaging. In this work, we aimed to select the lowest effective amount of nanoparticles into 4A z with antimicrobial activity.

## Materials and Methods

### Materials

Here, 4A z (Si/Al ≅ 2) was synthesized according to the formula below. Zinc acetate dehydrate, orthotitanate and ethanol were purchased from Merck. All the applied reagents were of analytical grade. It should be noted that in order to test antimicrobial activity, bacterial strains were obtained from Biological and Genetic Resource Center, Tehran, Iran. Nutrient broth and Nutrient agar were all purchased from Micromedia, Canada.

### Measurements

X-ray diffraction patterns (XRD) were performed using a Siemens D5000 diffractometer with Cu kα radiation (λ = 1.5418 A and 2θ = 4–70°) at room temperature, 40 kV and 30 mA.

The degree of crystallinity is measured by peak height method^46^ based on Eq. (1):

1 $${\rm{Crystallinity}}\,{\rm{Index}}({\rm{CrI}})=(\frac{{\rm{I}}-{{\rm{I}}}_{0}}{I})100$$

where CrI is crystallinity degree (%), I, is peak height at the angle of diffraction (2θ) related to crystalline section and I_0_ is peak height at the angle of diffraction (2θ) related to non-crystalline section.

Scanning electron microscope (SEM) (Philips XL30) was evaluated to catch SEM images and to carry out elemental analysis. The SEM sample was gold coated prior to examination and SEM was operated at 5 kV. MAP was obtained by wavelength-dispersive X-Ray spectroscopy (WDS) system to show the spatial distribution of elements in a sample and be extremely useful for displaying element distributions in polymer matrix, particularly for showing compositional zonation. Transmission electron microscopy (TEM) was determined by electrons microscope systems and was also used to indicate the interactions between the electrons and the atoms in order to observe crystal structure and features in the structure like dislocations and grain boundaries. The minimum inhibitory concentration (MIC_90_), minimum bactericidal concentration (MBC) and fractional inhibitory concentration (FIC) of MONP dropped into 4A z were also determined.

## Methods

### Preparation of 4A zeolite

4A z (alumina silicates) with a silica to alumina ratio (Si/Al ≅ 2) was synthesized by hydrothermal method. In this process, clinoptilolite and alumina were used as sources of silicon and aluminum, respectively, and then mixed with NaOH. In the final stage, the solution was conveyed to teflon reactor at 90 °C, in order to produce 4A z nanocomposite^37^.

### Preparation of ZnO doped into 4A zeolite

In this research, ZnO clusters were synthesized by the following stage response: in the first step, a solution of 0.17 g Zn(CH_3_CHOO)_2_·2H_2_O was prepared, mixed with a ratio of 5:95% w/w (nanoparticles:4A z) and was shaken for 30 min. In the second step, integration of zinc acetate to 4A z using an ion exchange process was done (24 h at 60 °C). Next, by the use of distilled water, the solution was washed and dried at 80 °C. The final powder was calcined at 500 °C for 2 h to produce ZnO/4A z nanocomposite.

### Preparation of TiO_2_ doped into the 4A zeolite

In this research TiO_2_ salt was created by the following stage response: in the first step, 0.2 g ortho titanate was dissolved in ethanol. Then, this solution was mixed with a ratio of 5:95% w/w (nanoparticles:4A z) and was shaken for 30 min. In the second step, integration of ortho titanate to 4A z using an ion exchange process was done (6 h at 90 °C). Next, by the use of distilled water, the solution was washed and dried at 80 °C. The final powder was calcined at 500 °C for 2 h in order to produce TiO_2_/4A z nanocomposite.

### Preparation of ZnO and TiO_2_ doped into 4A zeolite

In order to prepare ZnO/TiO_2_doped into 4A z, ZnO nanoparticle was prepared and mixed with a ratio of 2.5:95% w/w (nanoparticles:4A z). Then, 2.5% w/w titanium dioxide ethanol dried powder was added to the mixture of 4A z and ZnO and dried at 80 °C. The final powder was calcined at 500 °C for 2 h in order to produce TiO_2_ and ZnO/4A z nanocomposite.

### Determination of minimum inhibitory concentration (MIC), minimum bactericidal concentration(MBC) and fractional inhibitory concentration (FIC)

The antimicrobial activity of 4A z, TiO2/4A z, and ZnO/4A z was defined by micro dilution method on 96-well microplates according to previously reported methods (Tajik *et al*., 2015). Four bacterial suspensions including *Escherichia coli* O157:H7 (IBRC-M 10698), *Listeria monocytogenes* (IBRC-M 10671), *Pseudomonas fluorescens* (IBRC-M 10752), and *Staphylococcus aureus* (IBRC-M 10690) were collected after 24 h cultures on Nutrient broth and regulated to 0.5 McFarland standard turbidity (1.5 × 10^−8^ CFU/mL). In order to evaluating antimicrobial activity of nanoparticles, we used bacterial suspensions (20 μL), different concentrations of 4A z, TiO2/4A z, ZnO/4A z and TiO_2_, ZnO/4A z suspension (0.5, 1, 2, 3, 4, 5, 6 and 7 mg/mL), 20 μL for each nanoparticles or 4A z and 10 μL in combination; and 160 μL of Nutrient broth in tested wells. The last wells of micro-plates were considered as positive controls containing uninoculated broth with antimicrobials materials (nanoparticles), and as negative controls containing inoculated broth without antimicrobials materials. The microplates were incubated at 37 °C for 24 h under constant shaking (50–100 rpm) by a microplate shaker (Boeco, Hamburg, Germany). It should be noted that for *Pseudomonas fluorescens*, we needed 25 °C for 24 h. MIC_90_ values were determined as the lowest concentration with no visible bacterial growth. The best concentration of each antimicrobial agent was determined by MIC method.

The MBC is the lowest concentration of an antibacterial agent required to kill a particular bacterium^47,48^. It can be determined from broth dilution MIC tests by sub culturing to agar plates that do not contain the test agent. The minimum bactericidal concentration is identified by determining the lowest concentration of antibacterial agent that reduces the viability of the initial bacterial inoculum by ≥99.9%. The MBC is complementary to MIC; whereas, MIC test demonstrates the lowest level of antimicrobial agent that inhibits growth, MBC test demonstrates the lowest level of antimicrobial agent that results in microbial death^49,50^. The fractional inhibitory concentration was used for combination of materials based on the following equation:

$${{\rm{FIC}}}_{{\rm{A}}\& B}=\frac{{\rm{MIC}}({\rm{A}}\& B)}{{\rm{MIC}}({\rm{A}})+{\rm{MIC}}(B)}$$

where A refers to TiO_2_ nanoparticles and B refers to ZnO nanoparticles.

### Determination of antibacterial activity of nanoparticles doped in 4Azeolite

The disc diffusion test was performed to determine the antibacterial activity of nanoparticles doped in 4A z^51^. *Pseudomonas fluorescens*, *Staphylococcus aureus*, *Listeria monocytogenes*, and *Escherichia coli* O_157_:H_7_ suspensions were collected from 18 h nutrient broth cultures then adjusted to 0.5 McFarland standard turbidity (1.5 × 10^−8^ CFU/mL) and diluted (1:10) to the desired bacterial density (1.5 × 10^−6^ CFU/mL). The Mueller-Hinton agar medium was then inoculated with 0.1 mL of the bacterial suspensions (1.5 × 10^−6^ CFU/mL). Next, the suspensions of nanoparticles doped in 4A z and also 4A z individually, were prepared under sterile conditions and then situated on the surface of Mueller-Hinton agar plates (100 λ). The plates were incubated at 37 °C (for *L. monocytogenes*, *E. coli* O_157_:H_7_, and *S. aureus*) or 25 °C (for *P. fluorescens*) for 24 h, after which the inhibition zone around the discs were measured by a digital micrometer. Indeed, inhibition zone have shown the antibacterial effect of nanoparticles. The greater the diameter of the inhibitory zone, the greater antimicrobial activities of nanoparticles.

### Inductively coupled plasma mass spectroscopy (ICP-MS)

Migration tests were performed according to EU regulation for plastic materials and articles intended to be in contact with food (Regulation 10/2011/ EU)^52^. Migration of TiO_2_ and ZnO nanoparticles was assessed using a simulant (deionized water with pH = 6–7) over 12 days at 25–30 °C. Prior to the migration tests, the concentration of ZnO and TiO_2_ nanoparticles in 4A z was determined. In ICP-MASS assay, we first produced a nano bio-composite film with selected polymer and nanoparticles zeolite. Then, this nano bio-composite film was converted to food packaging by thermal processing. Next, to measure the migration of nanoparticles zeolite from food packaging to food product, we have used food simulant. Food simulant was filled in food packaging, packed and stored for 12 days. Next, the simulants were filtered through a 220 nm Millipore filter and then migrated nanoparticles were analyzed using ICP-MS (Agilent 7800 Quadrupole ICP-MS, Perkin Elmer, California, United States). In the final stage, the migration results were normalized to nanoparticles migrated/cm^2^ after 12 days. It should be noted that, on the first day, the measurement of nanoparticles zeolite in food packaging was conducted regardless of food simulant. This was to discover the exact amount of nanoparticles embedded in 4A z and polymer matrix.

### Ethical approval

This article does not contain any studies with human participants or animals performed by any of the authors.

## Results and Discussion

### Evaluation of XRD patterns

**Figure 1:**
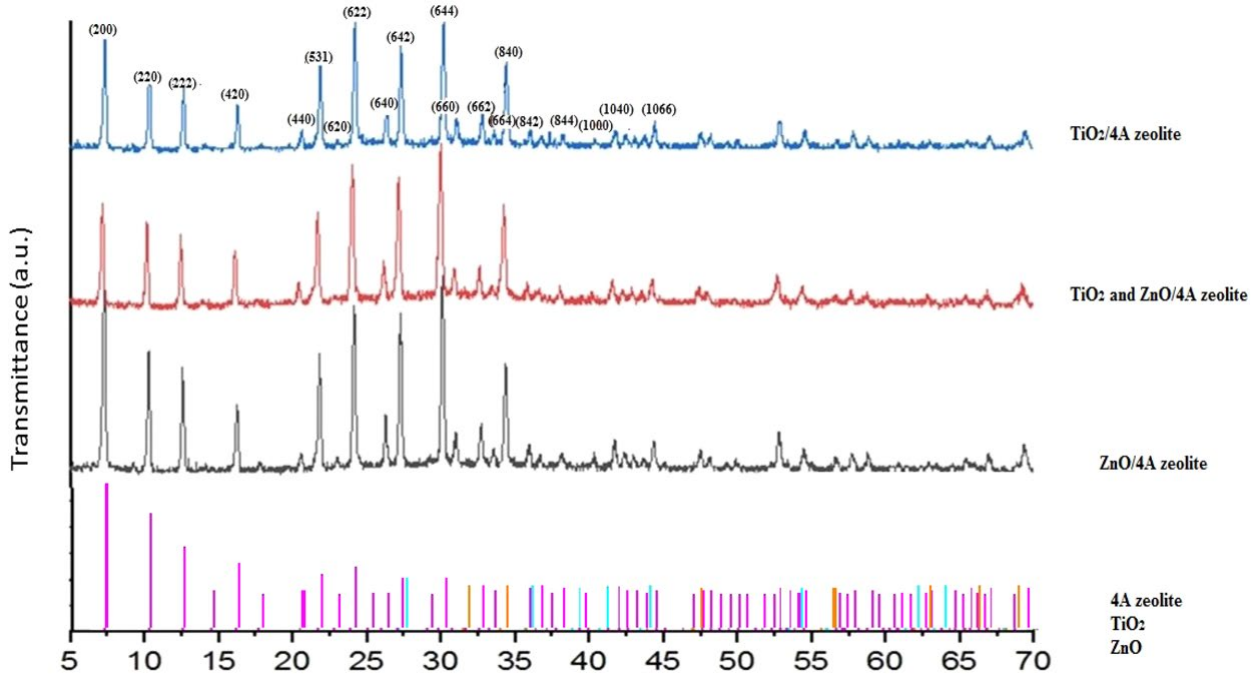
XRD spectra of nanocomposite TiO_2_/4A z, TiO_2_, ZnO/4Az and ZnO/4A z. Pink color is 4A z individually, blue color is TiO_2_ nanoparticle individually and orange color is ZnO nanoparticle individually.

XRD showed the impact of homogenization on crystalline structure of the combination^53,54^. XRD patterns of ZnO, TiO_2_, and ZnO/TiO_2_ supported into 4A z are shown in Fig. 1. Based on the obtained template, TiO_2_ nanoparticles in the form of anatase, had significant peaks at 2θ = 7.36, 10.31, 12.66, 21.90, 24.21,27.32,30.14, 30.19 and 34.41°. According to Scherrer equation (τ = κ λ/β cosθ), the average crystallite size of nanoparticle was approximated to be 50 nm. In this equation, τ is the average crystalline size, κ is the shape factor (about 0.9), λ is the wavelength of X-ray radiation, β is the full wide at half the maximum intensity and θ is the Bragg diffraction angle^55^. As shown in Fig. 1, the peaks of 4A z are visible at certain 2θ with high intensity. The diffraction peaks for ZnO and TiO_2_ were in good agreement with those given in the standard data (PCPDF, 79–0207 and JCPDS, 21–1272, respectively) with acceptable crystallinity. According to XRD patterns, it was found that ZnO and TiO_2_ was crystallized with hexagonal wortzite and anatase phase, respectively. On the other hand, it is clear to see that the width of the reflections is considerably broadened, which indicates a small crystalline domain size. In the spectra of oxide nanoparticles doped into 4A z, the presence of nanoparticles can be confirmed based on the observed peaks, in particular 2θ = 4–70. Regarding the significant peaks with miller indices of TiO_2_(642), (664) and ZnO (842), (664), they have considerable overlap with the peaks of 4A z (Fig. 1), and therefore, the oxide nanoparticle peaks are not clearly visible.

**Figure 2:**
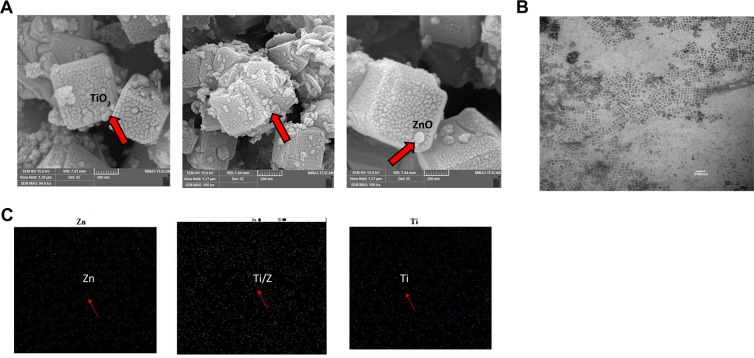
(**A**) Evaluation of SEM pattern; a: ZnO/4A z, b: TiO_2_, ZnO/4A z and c: TiO_2_/4A z. (**B**) Evaluation of TEM image; a: TiO_2_ and ZnO/4A z. (**C**) Evaluation of MAP images; a: ZnO/4A z, b: TiO_2_, ZnO/4A z and c: TiO_2_/4A z.

But according to MAP pattern, the presence of elements (titanium and zinc) has been confirmed which affects the intensity of 4A z peaks (Fig. 2). Also, it should be noted that in ZnO/TiO_2_ spectrum, the intensity of crystalline plates with Miller indices (200, 220 and 222) are lower than when there is only one type of metal oxide.

### Evaluation of SEM, TEM and MAP patterns

**Figure 3:**
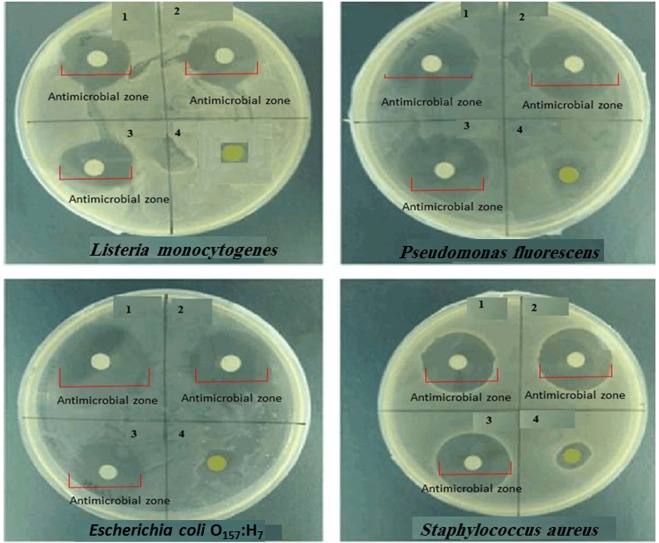
Disc diffusion test of nanoparticles doped in 4A z. In each figure: number 1 is ZnO/4A z, number 2 is TiO_2_/4A z, number 3 is ZnO and TiO_2_/4A z and number 4 is 4A z.

The size and morphology of the samples are illustrated in Fig. 2(A–C). According to Figs. 2A, 3A z particles with cubic structure are completely visible and ZnO and TiO_2_ nanoparticles are dispersed in the form of almost spherical particles into 4A z. Based on Fig. 2A, cube-shaped particles are completely apart from one another and the deformed structures are not visible. The image (Fig. 2A) showed that the matrix of ZnO and TiO_2_ nanoparticles had homogeneous and smooth surfaces without any roughness and cracks, which indicated proper synthesis of the materials. The size of 4A z particles, based on Fig. 2A,B, is 400 to 600 nm; besides the oxide nanoparticles are very thin and smaller than 50 nm. According to the obtained images (Fig. 2B,C), the dispersion of ZnO and TiO_2_ nanoparticles into 4A z structure is uniform and no accumulation has been observed. Also, cubic particles with square layers are present. Figure 2B shows a typical TEM micrograph of ZnO/TiO_2_ nanoparticles powder. This powder is formed by 10–50 nm size particles having equiaxed morphology. The particles are well separated from each other. Dark field imaging displayed that each particle was a single crystal. The nanoparticle sizes from TEM examination were in good agreement with both XRD and MAP.

### MIC, MBC and FIC values of nanoparticles

**Figure 4:**
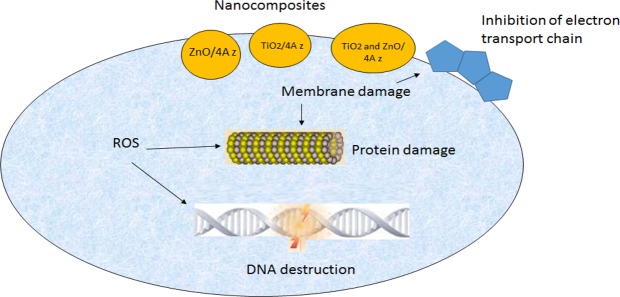
Antimicrobial mechanism of nanocomposites (TiO_2_/4A z, ZnO/4A z and TiO_2_ and ZnO/4A z).

MIC_90_, MBC and FIC values of 4A z, TiO_2_/4A z, ZnO/4A z, and TiO_2_, ZnO/4A z nanocomposite are shown in Tables 1–3. And 4A z do not have antimicrobial activity against bacteria’s^56^ (Table 1). TiO_2_/4A z, ZnO/4A z, and TiO_2_/ZnO/4A z nanocomposite were activated in the concentrations of 1–4 mg/mL against *E. coliO*157*:H*7*, S. aureus, P. fluorescens*, and *L. Monocytogenes*, separately. These results were in agreement with the outcome of prior studies^57,58^. The best concentrations of MIC were selected for TiO_2_/4A z, ZnO/4A z, and TiO_2_, ZnO/4A z nanocomposite as 2, 1 and 1 mg/mL, respectively; against microorganisms (Table 1). This concentration in MBC corresponded to 2, 2 and 2 mg/mL, respectively; and 0.25 mg/ml concentration of TiO_2_, ZnO/4A z nanocomposite against *E. coli O*_*157*_*:H*_*7*_, had an excellent FIC value (Table 3). Based on the results, gram negative bacteria were more sensitive compared to antimicrobial agents. Gram positive bacteria have a thick layer of peptidoglycan in their cell walls which have caused more resistance compared to gram negative bacteria against antimicrobial agent. Gram negative bacteria contains a thin peptidoglycan layer which facilitates the mobility of metal ion nanoparticles to the cell, and it also assists the interaction between nanoparticle and bacterial cell walls due to lack of thick peptidoglycan layer. Another aspect of gram negative bacteria is the negative charge of lipopolysaccharide layer. This charge has a role as a significant incorporation factor for positive ions, which consequently leads to nanoparticle emission, intracellular damages and destruction of DNA and proteins (Fig. 4).

**Table 1:** MIC of TiO_2_, ZnO, and TiO_2_ and ZnO doped in 4A z against bacteria.

Bacteria	MIC_90_ (mg/ml)			
	4A z	TiO_2_/4A z	ZnO/4A z	TiO_2_/ZnO/4A
*E. coli* O157:H7	0	2 ± 0.01	2 ± 0.02	1 ± 0.01
*S. aureus*	0	3 ± 0.01	3 ± 0.02	2 ± 0.01
*P. fluorescens*	0	2 ± 0.01	1 ± 0.01	1 ± 0.01
*L. Monocytogenes*	0	3 ± 0.01	2 ± 0.00	2 ± 0.01

MIC: Minimum Inhibition ConcentrationTiO_2_: Titanium dioxide, ZnO: Zinc oxide, 4A z: 4A zeolite.

**Table 2:** MBC of TiO_2_, ZnO, and TiO_2_ and ZnO doped in 4A z against bacteria.

Bacteria	MBC (mg/ml)			
	4A z	TiO_2_/4A z	ZnO/4A z	TiO_2_/ZnO/4A z
*E. coli* O157:H7	0	2 ± 0.01	3 ± 0.02	2 ± 0.01
*S. aureus*	0	4 ± 0.01	4 ± 0.01	3 ± 0.01
*P. fluorescens*	0	3 ± 0.01	2 ± 0.01	2 ± 0.01
*L. Monocytogenes*	0	4 ± 0.02	3 ± 0.00	3 ± 0.01

MBC: Minimum bactericidal ConcentrationTiO_2_: Titanium dioxide, ZnO: Zinc oxide,4A z: 4A zeolite.

**Table 3:** FIC of TiO_2_/ZnO doped in 4A z against bacteria.

Bacteria	FIC (mg/ml)
	TiO_2_/ZnO/4A z
*E. coli* O157:H7	0.25 ± 0.01
*S. aureus*	0.33 ± 0.01
*P. fluorescens*	0.33 ± 0.01
*L. Monocytogenes*	0.40 ± 0.01

FIC: fractional inhibitory concentration TiO_2_: Titanium dioxide ZnO: Zinc oxide, 4A z: 4A zeolite.

### Antibacterial activity of nanoparticles doped in 4A zeolite

The disc diffusion procedure was conducted to assess the antimicrobial activity of nanoparticles doped in 4A z (Table 4). Inhibition zone diameter indicated that TiO_2_, ZnO/4A z had a greater antimicrobial effect against bacteria compared to the combination of individual nanoparticles with 4A z (Fig. 3). Also it should be noted that, 4A z individually do not have an antimicrobial activity against bacteria^56^. Based on the results, Gram-negative bacteria (particularly *E. coli* O_157_H_7_) were more sensitive to the antimicrobial agents. The main purpose of using 4A z, as a carrier with nanoparticles, is to control the release of them and increase the antimicrobial activity. The highest zone of inhibition was seen against *E. coli* O_157_H_7_ including_;_ 6.86 ± 0.03 mm, 9.13 ± 0.03 mm, and 10.73 ± 0.04 mm for ZnO/4A z, TiO_2_/4A z and ZnO and TiO_2_/4A z, respectively; while *S. aureus* had the lowest inhibitory zone in all treatments (*P* < *0.05*). Indeed, nanoparticles disturb the permeability of both the cell wall and cell membrane, thereby affect biomolecules such as DNA and protein, and prevent processes such as DNA replication and protein synthesis^59^.

**Table 4:** Disc diffusion analysis of nanoparticles doped in 4A z.

Bacteria strains	Control sample	Inhibition zone (mm)		
		Treatment 1	Treatment 2	Treatment 3
*L. monocytogene*	0	6.25 ± 0.02 a	8.82 ± 0.03 e	9.51 ± 0.01 i
*E. coli* O_157_;H_7_	0	6.86 ± 0.03 b	9.13 ± 0.03 f	10.73 ± 0.04 j
*S. aureus*	0	6.21 ± 0.02 c	7.58 ± 0.6 g	9.22 ± 0.02 k
*P. fluorescens*	0	6.34 ± 0.03 d	8.97 ± 0.04 h	9.85 ± 0.01 l

Control sample: Zeolite 4A, Treatment 1: ZnO/4A z, Treatment 2: TiO_2_/4A z, and Treatment 3: ZnO and TiO_2_/4A z.

Date are presented as mean ± SD and analyzed with one-way analysis of variance. Different letters represent statistical significance among different treatment using the Tukey.

Titanium dioxide (TiO_2_), is also called titania, is one of the most widely used semiconductor nanoparticles with specific hydrophilic and photocatalytic properties, which caused to antimicrobial and ultraviolet (UV) protecting characteristics^51,60^. These nanoparticles are extensively used in producing polymer nanocomposites in food packaging. TiO_2_ nanoparticle works in two phases: first, the ability to degrade bio polymeric compound (such as polysaccharides and proteins)^61^ on its surface and second, to alter the surface properties of the objects to hydrophilic state when TiO_2_ is placed on that surface. In Europe, TiO_2_ (E171) is confirmed and categorized as a color additive in confectionaries, dairy products, and soft drinks under Directive of 1994/36/EC^62^. TiO_2_ is more prone to oxidization when exposed to ultraviolet (UV) in a wavelength lower than 385 nm. As a result, TiO_2_ can produce active oxygen species when exposed to sunlight^63^. Furthermore, the antimicrobial activity of TiO_2_ is related to its crystal structure, the kind of artificial light, UVA light intensity, shape and size^64^ as well as production of ROS, active radical species, hydrogen peroxide, superoxide radical, and hydroxyl radical^64–66^. These active species destroy the outer membrane of the bacteria, namely phospholipids, proteins and lipopolysaccharides and finally damage the bacteria.

ZnO is recognized as a colorless, wide band gap semiconductor with an optical band gap in UV region which makes it applicable as an impressive absorbent of UV radiation. Currently, ZnO is listed as generally recognized as safe (GRAS) by the US Food and Drug Administration (FDA) and is consumed as a food additive, considering the fact that zinc is an important trace element in nutrition^67^. The antimicrobial activities and potential applications of ZnO nanoparticles in food preservation have been approved^68^. In this regard, ZnO nanoparticles were combined in polymeric matrix in order to supply the packaging material with antimicrobial activity and to improve some packaging properties^69,70^. Furthermore, the antimicrobial properties of ZnO is related to photocatalytic activity of H_2_O_2_. Both Zn^+2^ and ZnO particles hold antibacterial activities. The antimicrobial activities of ZnO at nanoscale would yield affordable and safe innovative strategies^69,71^. Moreover, ZnO has been blended into the linings of food cans used for meat, fish, corn, and peas to preserve colors and hinder spoilage because of its antimicrobial action.

Indeed nanoparticles have the ability to decline or remove the microorganism resistance by two mechanisms: (a) free metal ion toxicity arising from dissolution of metals from the surface of nanoparticles and (b) oxidative stress via generation of reactive oxygen species (ROS) using hydrogen peroxide (H_2_O_2_) and organic hydro peroxides (OHP) on the surface of nanoparticles^72^. Actually, nanoparticle can effect on the survival of microorganisms by agglomeration on the surface of bacteria and alter the structure of lipids, peptidoglycan, proteins and their DNA^73^. But there may be differences in the impact of nanoparticles on types of specific microorganisms; for instance, Sierra *et al*.^74^ showed a higher antimicrobial effect against *Streptococcus mutans* of Cu nanoparticles at lower concentrations than zinc. In another study, Ruparelia *et al*.^75^ showed that copper nanoparticle has great impact as an antimicrobial agent against *E. coli*, *Bacillus subtilis*, and *Staphylococcus aureus* in comparison with TiO_2_. Our results are in agreement with those of Lou *et al*.^59^ and Yael N *et al*.^76^.

### Inductively coupled plasma mass spectroscopy

Migration is a mass transfer process where low molecular mass particles are released into the surroundings. To assess migration, the system must be simplified and particles must be analyzed separately. The package is assumed to be homogeneous, the food is substituted by food simulants and the substance is introduced at the recognized concentration. In this research, nanoparticles ion releasing capacity in food simulants of the composites was quantified by ICPMASS.

Zinc and Titania ions are doped into 4A z through ionic exchange, preferably with Na^+^ ions^77^. Next, ions are released from composites through ionic exchange with cations/protons present in food simulants, and their concentration determines the amount of released nanoparticles. Therefore, for applications where a long-term antibacterial activity is expected, nanoparticles zeolites should be placed in solutions with low ionic strength^78^.The immersion of matrix (nanoparticles doped in 4A z) in distilled water (as a food simulant) demonstrated that nanoparticles were sensitive to water. When the matrix was immersed in water for a long period of time, the water molecules interacted with polar groups in nanoparticles were doped in 4A z, which led to the swelling and deformation of the matrix. According to ICP-MS results (Table 5), the amount of ZnO in ZnO/4A z was initially 1031 ppb, and decreased to 943.3 ppb (ZnO/4A z) after 12 days of immersion in food simulant. These values were 655 and 3.1 ppb for TiO_2_/4A z, respectively; while they were recorded to be 1259 and 344.1 ppb for ZnO and TiO_2_/4A z. Based on the results, the migration values for ZnO, TiO_2_, and ZnO and TiO_2_ were approximately 47%, 0.47% and 21%, respectively. It is worth mentioning that ZnO and TiO_2_ have been approved by the US Food and Drug Administration (FDA) as safe compounds to be used in food and food contact materials at restricted quantities below 2 and 1% of the food weight for ZnO and TiO_2_ nanoparticles, respectively^79,80^. Moreover, low amounts of ZnO and TiO_2_ nanoparticles are beneficial for the human body because of their antimicrobial and anticancer properties. ICP-MS was used to detect the metal ions with high sensitivity, and it could detect very low signals of ZnO and TiO_2_ nanoparticles from the digested solutions. These results revealed that the slight migration of nanoparticles occurs in food simulant; however,this amount is in accordance with the standard rate by FDA^81^. Our results regarding TiO_2_ nanoparticles are in accordance with those of Lian *et al*.^82^.

**Table 5:** Migration assay of nanoparticles zeolite with ICP-MASS test.

Nanoparticles zeolite	ZnO/4A z	TiO_2_/4A z	ZnO and TiO_2_/4A z
Migration rate (ppb)			
Frist day	1031	655	1259
Twelfth day	943.3	3.1	344.1

## Conclusions

TiO_2_, ZnO and TiO_2_/ZnO nanoparticles supported into 4A z were successfully synthesized through hydrothermal method, and were evaluated for antimicrobial activity for the first time. The size of 4A z particles were around 400 to 600 nm and the average crystallite size of nanoparticles was approximately 50 nm. The XRD, SEM, MAP and TEM analysis demonstrated that crystallographic plate, size, shape and morphology of nanocomposite (nanoparticles with 4A z) are very close to 4A z itself. Moreover, the results of antimicrobial test confirmed a considerable antimicrobial activity against gram positive and negative bacteria of the nanoparticles supported in 4A z. Indeed, the antimicrobial activity of nanoparticles embedded into 4A z was dependent on the type of nanoparticles and on the species of microorganism (gram positive or gram negative). The most sensitive bacteria were *P. fluorescens* and then *E. coli* O157:H7. Embedding of nanoparticles in 4A z caused to control the release of them, and enhance their antimicrobial properties. Hence, using these composites can be a promising approach to create new active packaging in food industry.

## Supplementary information

Supplementary information

Supplementary information

Supplementary information
