# Supplementary figures and images for: Antimicrobial activity of Titanium dioxide and Zinc oxide nanoparticles supported in 4A zeolite and evaluation the morphological characteristic
Source: Sci Rep. 2019 Nov 25;9:17439. doi: 10.1038/s41598-019-54025-0 (PMC6877518; doi:10.1038/s41598-019-54025-0)

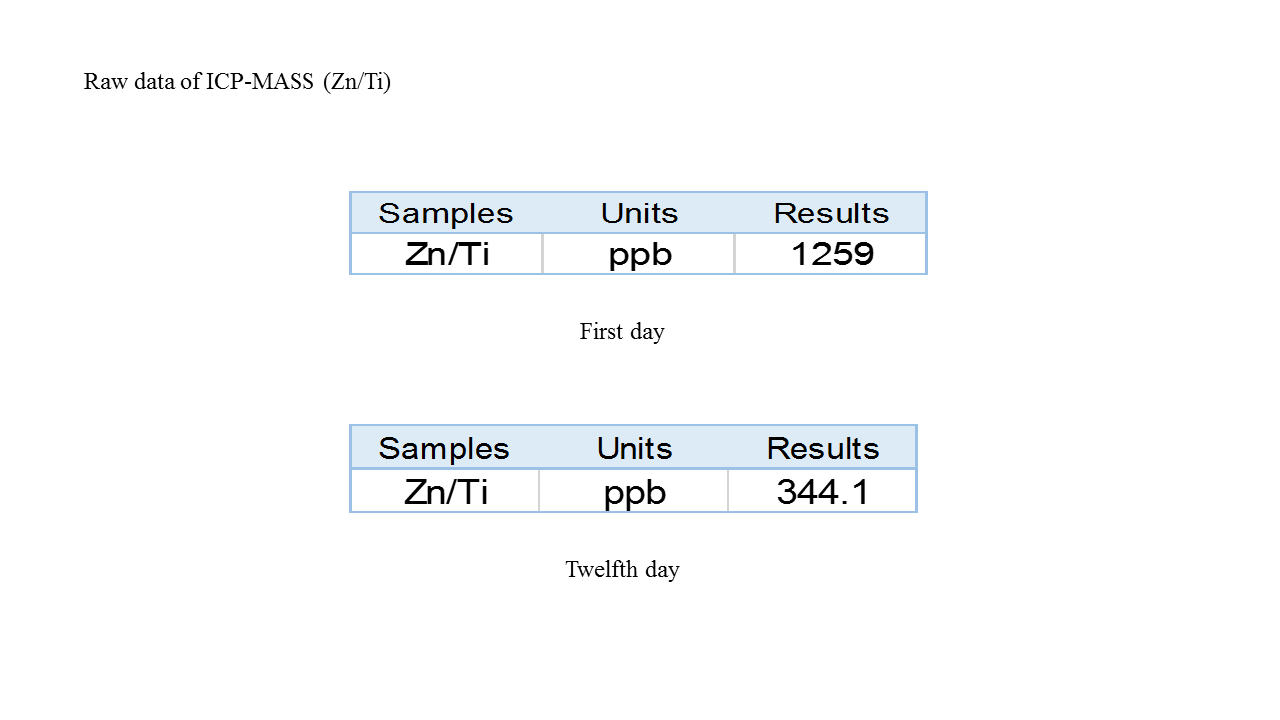

Supplement: Supplementary file 1 — Supplementary information [file 41598_2019_54025_MOESM1_ESM.tif]

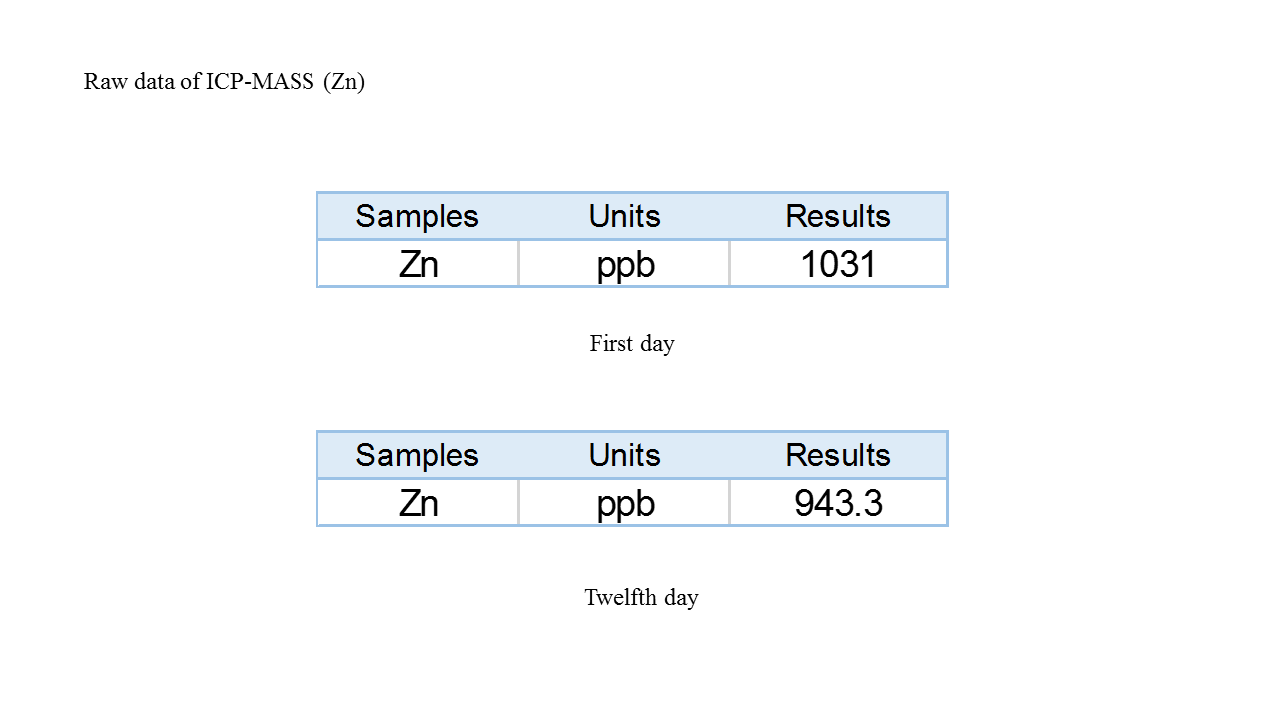

Supplement: Supplementary file 2 — Supplementary information [file 41598_2019_54025_MOESM2_ESM.tif]

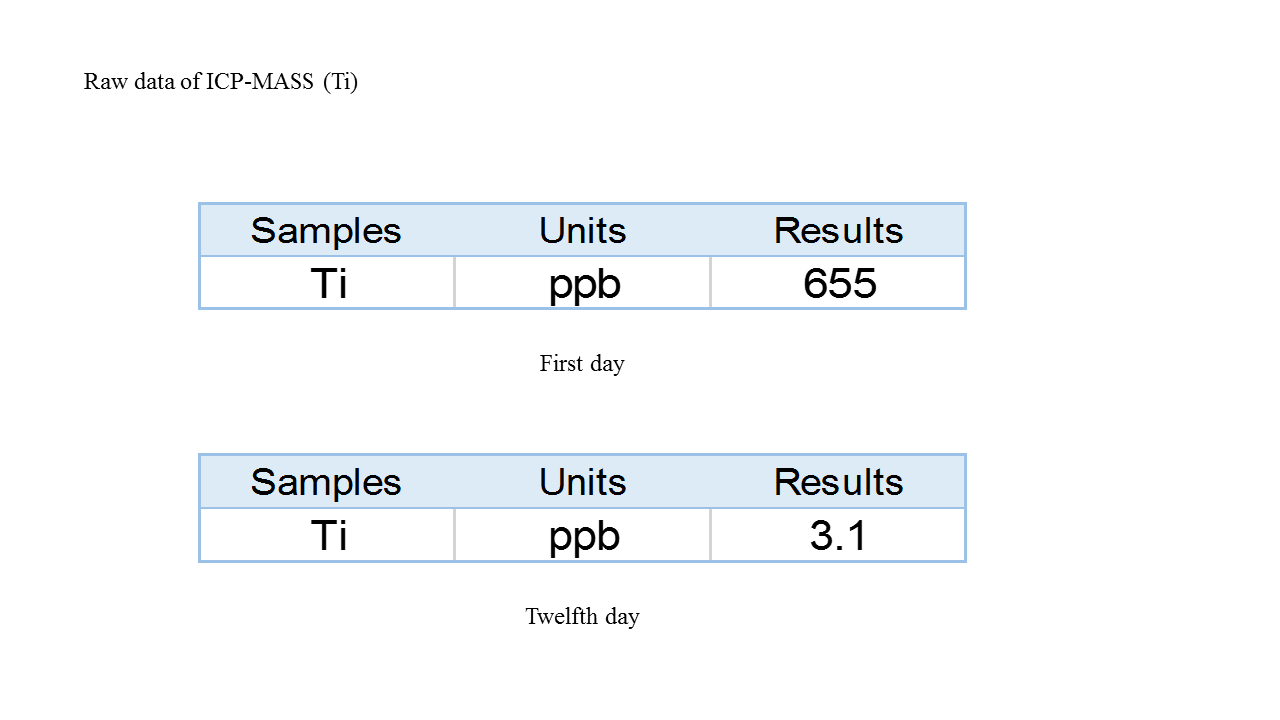

Supplement: Supplementary file 3 — Supplementary information [file 41598_2019_54025_MOESM3_ESM.tif]
